# Supplementary material for: From decision to action: Detailed modelling of frog tadpoles reveals neuronal mechanisms of decision-making and reproduces unpredictable swimming movements in response to sensory signals
Source: PLoS Comput Biol. 2021 Dec 13;17(12):e1009654. doi: 10.1371/journal.pcbi.1009654 (PMC8699619; doi:10.1371/journal.pcbi.1009654)
Supplement: S6 Table — (DOCX) [file pcbi.1009654.s008.docx]

| tIN to dIN EPSPs | | |
| --- | --- | --- |
| AMPA+NMDA | model | exp |
| Amplitude (mV) | 2.2 | 2.6 |
| Duration at 50% amplitude (ms) | 13.8 | 14 |
| Time to peak (ms) | 3.4 | 5.2 |

Supplementary Table 6: Measures of single *tIN* to *dIN* EPSPs in model simulations and experiment. Experimental measures (exp) have been reported in Buhl et al., 2012. We show measures a mixture of AMPA and NMDA components. These measures are: the average of the maximal EPSP amplitude, the EPSP duration at 50% amplitude, and time to peak from the stimulus. The experimental values were analyzed in pairwise (*tIN,dIN*) recordings. Strengths of the model AMPA and NMDA synapses are $w_{AMPA}=0.35nS$ and $w_{NMDA}=0.3nS$, respectively. The post-synaptic *dIN*s are electrically coupled to other 10 *dIN*s with electrical coupling 0.2nS.
