# Supplementary material for: From decision to action: Detailed modelling of frog tadpoles reveals neuronal mechanisms of decision-making and reproduces unpredictable swimming movements in response to sensory signals
Source: PLoS Comput Biol. 2021 Dec 13;17(12):e1009654. doi: 10.1371/journal.pcbi.1009654 (PMC8699619; doi:10.1371/journal.pcbi.1009654)
Supplement: Attachment — Submitted filename: revision summary table_FINAL.docx [file pcbi.1009654.s009.docx]

Dear Editors,

Thank you for consideration of our paper for publication in PLOS Computational Biology.

We are grateful to anonymous reviewers for their valuable comments and advice for improving the quality of the manuscript. We revised the manuscript following the comments from the reviewers. In summary, the manuscript is now 43 pages long (39 pages long before submission), two new figures in the methods section, several additional references and corrections of the text and figures. The revision is explained in more details in the following table.

**REVIWER #1**

| Page 2. In the intro paragraph describing the lamprey model, consider another way to describe that model then "The model". It can lead to confusion as to whether you are describing the lamprey model or the model described in this study | Done. Also, we adjusted description of lamprey modelling, made it shorter and clear. We also added several lines about lamprey modelling to Discussion. |
| --- | --- |
| Page 3. 3rd paragraph. I don't think you can answer the questions with a model but you can certainly propose answers or theories to test | At the end of this paragraph we added the following: “Results of our “whole” animal modelling propose answers and make predictions for experimental testing”. |
| Figure 2B-D. There is a mismatch between the brown in the spike times and the brown in the model (which looks more off-gray than brown). | The figure has been adjusted. |
| Page 8, last paragraph. Replace The estimate with To estimate | Corrected |
| Page 11. The phrasing Preliminary recordings implies that these are experimental recordings | We have inserted explanation of “preliminary EXPERIMENTAL recordings” to make the text clear and avoid confusions. |
| Page 12. Last sentence. By "similar percentage" you mean same percentage of swimming and no swimming response? | We have re-written this sentence to clarify the result of selection the connectivity probability for hexINs. |
| Can you clarify whether panels g-j from Figure 4 are from the same CNS model or from different CNS models? It would be useful to display the range of left and right membrane potential time courses that preceded each type of event (Swim, No swim, synchrony and one-sided) to get a sense of the sensory memory that usually leads to a specific event | Panels in Fig. 4 g-j show results of repetitive simulations with the same stimulus on the left side. For each simulation we randomly select the connection architecture and connection strengths. Response to stimulus depends on the time course of average membrane potential on the left and right side (shown by red and blue curves). We adjusted the text to make this important matter clear. |
| "Remarkably, mapping these CPG spike trains to the VT model muscles results in reliable swimming behaviour similar to real tadpole." Was there any parameter fitting in the VT model to make the tadpole model swim like a real tadpole? If there was, then i am not sure it is remarkable. But if the model was created independent of the CNS model output and it worked within the first instance of feeding the VT model with the CNS model, then that is remarkable indeed | We created VT biomechanical model independently of CNS model using as many biological measurements as possible to design the model and prescribe parameter values. Of course, several parameter values were not available and we adjusted them. The main parameter of the VT model which was fitted is the rigidity (or elasticity) of the tadpole body (more precisely, rigidity coefficients for the notochord, muscles, belly and for the rest body tissue), which are now described in a new section of METHODS. Another important parameter is the maximal contraction force produced by tadpole muscle segments. It was also chosen to provide realistic movement – to cause the model to perform body bends with the amplitude close to the real one. Indeed, the VT model may be described quite well with the sentence: The model was created independent of the CNS model output and it worked within the first instance of feeding the *VT model* with the CNS model. We also added a new line to the paragraph highlighted by the reviewer to make this clear. |

Reviewer #2: General comments

| Although the authors make a statement throughout the manuscript that the sensory pathways of the model control motor behavior, this statement seems misleading. Yes, sensory pathways originating from skin touch pathways evoke motor responses or stop them, but once a motor response started, it is controlled by feedforward commands from the CPG and motoneurons. In fact, the model lacks motion-dependent sensory signals that have been shown to be critical for performing coordinated movements (e.g., Pearson et al., Trends in neurosciences 29: 625-631, 2006; Bacque-Cazenave et al., J Neurophysiol 113: 1772-1783, 2015). Perhaps the proprioceptive feedback can be incorporated in the model in the future, but this issue should be discussed. | We would expect swimming movements to excite Lateral line and Gabaergic Kolmer Agdur cell firing but don’t know anything about whether these sensory neurons affect the motor system. At present there is no evidence for any specific type of motion sensitive proprioceptors providing feedback about movement. This is unexpected and remarkable! But, this is a very simple animal at an early stage of development.  We have addressed this matter in the Discussion by adding a new paragraph and several references (including two references suggested by the Reviewer #1): “. The hatchling *Xenopus* tadpole was chosen for study because, compared to most animals, its nervous system and behaviour is remarkably simple at this very early stage of its life. In most animals, locomotion is not a simple feedforward process coordinated by the CNS but relies critically on motion-dependent proprioceptive sensory feedback signals…” |
| --- | --- |
| Another weakness is that the description of the musculoskeletal system does not have the same rigor and level of details as of the nervous system. Specifically, it seems important to provide details or references of basic properties of the muscle model (the force-length and force-velocity relationships of the contractile component, the force-length relationship of the series and parallel elastic components, history-dependent characteristics, i.e. force enhancement and depression during muscle stretch and shortening), muscle architecture (orientation of muscle fibers with respect to the tendon, muscle fascicle and tendon length, physiological cross-sectional area, muscle moment arms, etc.). All these characteristics affect motor behavior and thus should be described in more details, so other researchers are able to reproduce the simulations. | To build our VT model we construct a detailed 3D tadpole body where the body shape matches experimental measurements and compose the tadpole body and surrounding water from many small “particles” of different mass. Particles are connected to each other by “springs”. Stiffness of springs was selected to reflect the elastic properties of body tissues. Therefore, basic muscle properties are not defined during the VT model design. To find these properties we have performed numerical experiments with the VT model to measure (empirically) basic properties of the muscle model: (1) contraction velocity vs force (load) and (2) muscle tension vs muscle length. Although our approach to muscle modelling is quite simple and straightforward, the output was similar to experimental data for real *X. laevis* muscles (Jaspers et al., 2004). Descriptions of simulations to find these basic muscle properties along with description of VT muscle and body architecture were added in METHODS section. |
| Figure 1c: The mode of motion control appears to be feedforward without motion-dependent feedback from moving body that modulates CPG activity. This seems counterintuitive given that motion-dependent feedback is critical for maintaining coordinated locomotion (see works of Pearson, Duysens, Prochazka, Akay and others). | We have already replied to the feedback question above (responses to Reviewer #1 comments:  We would expect swimming movements to excite Lateral line and Gabaergic Kolmer Agdur cell firing but don’t know anything about whether these sensory neurons affect the motor system. At present there is no evidence for any specific type of motion sensitive proprioceptors providing feedback about movement. This is unexpected and remarkable! But, this is a very simple animal at an early stage of development.  We addressed this matter in Discussion by adding a new paragraph and several references (including two references suggested by the Reviewer #1): “. The hatchling *Xenopus* tadpole was chosen for study because, compared to most animals, its nervous system and behaviour is remarkably simple at this very early stage of its life. In most animals, locomotion is not a simple feedforward process coordinated by the CNS but relies critically on motion-dependent proprioceptive sensory feedback signals…” |
| Page 6, paragraph 2, “swimming behavior controlled by skin sensory inputs”: controlled or initiated/stopped? The term control would imply that the ongoing rhythmic activity is constantly modulated by skin sensory inputs. | As mentioned in the previous response we do not consider sensory modulation during ongoing swimming due to lack of evidence in young tadpoles. We changed the referred text to: “This model can generate a complete sequence of swimming behavior initiated and stopped by skin sensory inputs” |
| Page 6, paragraph 2, “cycle period lengthens”: What does cause this period lengthening? | This is due to NMDA depression in *dIN* recurrent connectivity. We have adjusted the text to explain this effect. As swimming continues, cycle period lengthens due to NMDA depression in *dIN* recurrent connections (see also section “OPERATION OF CNS MODEL: SWIMMING INITIATION” below). In addition, swimming can be accelerated by a trunk skin stimulus. Fig. 2a (bottom panel) shows the dynamics of period length [19, 39, 40]. |
| Page 8, paragraph 1, “2 sensory, 2 sensory pathway”: ? | We adjusted this sentence to make a clear explanation of added populations. “Here we extend our previous swimming model [19] by adding two populations of sensory neurons (*tSt* and *tSp)*, two sensory pathway neuronal populations (*tIN* and inhibitory *MHR*) and sensory memory neurons (*exIN*s)”. |
| Page 9, paragraphs 2 and 3: Is there a way to independently verify the distribution of connections in the animal? | Anatomical measurements of neuronal characteristics and multiple pair-wise recordings can be used to clarify the distribution of connection probabilities. Currently, available measurements and pairwise recordings between sensory pathway and CPG neuronal populations are limited to verify these distributions. Therefore we consider connection probabilities and their distributions as modeling predictions for experimental testing.  To clarify this point we added a short explanation in Discussion. |
| Figure 4k: Is the maximum value on the vertical axis 0.9% or 90%? | It is 90%, sorry. Thank you for spotting this mistake, which we corrected. |
| Page 18, paragraph 2, “striving for a balance between model accuracy and computational performance…”: How closely the mechanical properties of the model match those of the tadpole's body? | We build the biomechanical model using as many biological measurements as possible to design the body shape and prescribe parameter values to interactive particles and muscles. Of course, increasing the number of particles (and springs) increases time of simulations. Therefore, we find a balance between the number of model elements and time of computations.  To illustrate our approach and matching VT model to real tadpole: We used the actual densities of the tadpole belly (1060 kg/m^3^) and the rest body (1035 kg/m^3^) known from experiment (Roberts, A., Hill, N. A.  and Hicks, R. (2000) Simple mechanisms organize orientation of escape swimming in embryos and hatchling tadpoles of *Xenopus laevis*). Selection of these values resulted in swimming velocity of the tadpole model which differs from real tadpole swimming velocity by just 8% (19.3 mm/s vs 21 mm/s, which is an indirect evidence of the quality of the model.  To clarify this matter we added a new section to METHODS. |
| Page 18, paragraph 3, “contraction dynamics based on adult from muscle”: What are contractile properties of the muscles included in the model? Are a series and parallel elastic component are included? | All basic properties of the VT muscles are now described in the new section of METHODS. For example, force-length and force-velocity relationships diagrams were obtained in several numerical experiments. |
| Page 20, paragraph 4, “Using recorded soma and dendrite locations…”: This is an elegant method of finding neuronal connections. But it's not clear if the generated connections are biologically realistic and match the real ones. This should be clarified. | Yes, our developmental method is “biologically realistic” because statistical characteristics of neurons and connections generated by the developmental model are very similar to statistics of experimental data which are measured and recorded from real tadpoles. We adjusted text in Discussion to clarify this point. |

Reviewer #3:

| The text is quite difficult to follow and I recommend careful proofreading for clarity, but also for typos and grammar. Generally, I had the impression of carelessness in your text due to the many errors (especially article use, plural/singular, punctuation) | Sorry for this mess. The revised text has now been checked by native English speakers. |
| --- | --- |
| I found it difficult to assess the experimental basis for your proposed exINs just from your text. From a quick survey of one of the references you give, I only found some information in reference [37]. I assume your exIN neurons correspond to the proposed hexNs in that paper. Why the name change? From there I gather that these neurons are still unidentified and your model now proposes characteristics of an input population that could provide a long-lasting activity in response to short sensory stimuli. Please revise your text to make clear what experimental data exists and what are your model assumptions. To illustrate what I mean: You write on page 3 that ”We focus on the critical role of exINs which provide a basic sensory memory of the brief stimulus”. This sounds to me as if these neurons have been identified and characterized in their role. If that is true I couldn’t find it in your references (see also my comment on references). I In the Methods you state that “few experimental recording of these cells are available”. I might be missing this in ref [37] could you point me and the reader in the right direction? | Experimental basis of exINs has been discussed in two papers: [37] (Koutsikou et al., 2018, named “hexN”) and [20] (Roberts et al., 2019, named “exN”). We follow your recommendation to unify the name and now in our paper we use the call the sensory memory neurons “hexN”, because they are in the hindbrain and they are extension Neurons.  We have inferred the *hexN* activity from the *hdIN* post-synaptic potentials [37] and in addition we have presented examples of candidate *hexN* recordings in [20] (also two recordings are shown in Fig. 4d of our current paper).  We added a short explanation in Section “Role of sensory memory neurons…” and a reference to recent publication on this topic (Messa and Koutsikou 2021, biorxiv). The dissertation by Lisa James (ref [36]) is publicly available from The University of Bristol library but, probably, as a paper copy only. We now have asked permission to make this thesis online in the University of Bristol “Pure” system and we the request is currently waiting in the pipeline. If necessary we can send the thesis to the reviewer/editor via email in electronic copy.  These sensory memory neurons (*hexN*) are still not properly characterised which would require many more whole-cell recordings. In the Introduction we say: We focus on the critical role of *hexN*s which we propose provide a basic sensory memory of brief skin “touch” stimuli (Koutsikou et al., 2018; Roberts et al., 2019). |
| Throughout the text, please double-check your references. I didn’t follow all references, but a few that I checked didn’t seem appropriate. For example: a. Page 2, 1st para: the selection of one review [1] and one original article [6] seems random to me. The review is focused on spinal locomotor neuronal populations and the article describes a brainstem population that stops locomotion. Could you include more examples? b. Page 2, 2nd para: “The model of hatchling Xenopus tadpole is built at a unique level of detail compared with existing models”. Reference [7] doesn’t seem at all related to the level of detail that has been used for models of locomotor systems. c. Page 3, 4rd para: reference {22} doesn’t support the statement that “exINs provide a basic sensory memory”. d. Page 4, is the thesis publicly available? I couldn’t get access. | We have checked, adjusted references, added additional proper references.  Ref [1] and [6]: we added appropriate references.  Instead of [7] we inserted references to several recent models of locomotion (mice, zebrafish, and lamprey).  We deleted ref [22] and adjusted text: We focus on the critical role of *hexN*s which we propose provide a basic sensory memory of brief skin “touch” stimuli (Koutsikou et al., 2018; Roberts et al., 2019)  Reference to the thesis was deleted. |
| Page 1, abstract: “reveals that hindbrain sensory memory populations on each side compete to initiate reticulospinal neuron firing and start swimming.” I don’t think you can state it that strongly. Your model predicts that these neurons could play the role, but it doesn’t show anything conclusively. | Agree. Adjusted text: In response to skin stimulation, it generates realistic sensory pathway spiking and shows how hindbrain sensory memory populations on each side can compete to initiate reticulospinal neuron firing and start swimming., |
| Page 3, 5th para: Caenorhabditis is misspelled, elegans is missing | corrected |
| Page 4, 2nd para: you seem to be using hdINs, dINs, and hindbrain dINs interchangeably. I suggest consolidating to a consistent nomenclature. If hdINs and dINs are distinct populations, please clarify that throughout the text and in your schematic. | Agree. We inserted a note to explain this. |
| Figure 2, make sure colors between the schematic and recordings match. For example, I found it difficult to find the corresponding recording for dINs since the shades of brown are so different. | The colours of the schematics have now been adjusted for better matching with recording colours. |
| Figure 2, add (RC) after “rostro-caudal” | Done |
| Page 8, 3rd para: “one of three ways” implies these are alternatives. I think you applied all three instead. If I’m misunderstanding, please explain how you are using these in alternative ways and what the outcomes are. | We changed the text to “The connections between *hexN*s and other populations are assigned in as follows:...” |
| Page 8, 5th para: “Remarkably, when different connectomes were projected to the physiological model of spiking neurons, all of them generated functional behavior that correspond to the ones found in experiments and described below.” Please mention here briefly under what constraints this is true. | The structure and all parameter values of the physiological model, which generates functional behavior, are fixed. To run the physiological model we generate a connectome (adjacency matrix of connections) and “project” it to the physiological model. To get the connectome we use the algorithms which are described in the paper. These algorithms include some random components, therefore, if we repeat the algorithm with unchangeable parameter values, the resulting connectome will differ from all others. We generated many (about 1000) connectomes and, remarkably, all of them produced responses corresponding to the ones found in experiments and described in this paper. We added a short explanation and also new lines to the discussion to clarify this further. |
| Page 9, 2nd para: change “formulas” to “equation”, also for other instances of “formula” in the text | Done |
| Figure 3a: label axes with “presynaptic populations” and “postsynaptic populations” | We adjusted figure adding these labels |
| Figure 3b: the inversion of pre and postsynaptic axes in comparison with 3a is confusing. Maybe also label with pre and post in addition to your explanation in the text. | We followed the suggestion and labelled pre and post in figure 3b |
| Figure 4a: are these 6 trials of the same neuron? Six different neurons? | These 6 recordings are from the same *hdIN*. We adjusted caption to the figure. |
| Figure 4k: It looks as if none of the exIN probabilities were able to generate all four conditions. Is that correct? Could you comment on that, and I think it's also worth mentioning this in the text. | Actually, for the lower hexN commissural connection probability p~0.25 in model simulations we observed 4 possible cases. We added the note to the text. |
| For Fig 4g-j, what probabilities did you use there? | We used p=0.33 (optimal probability) for simulations shown in Fig. 4g-j. We adjusted the text to explain this. . |
| When you say on page 12 "we selected the optimal value of exIN commissural connection probability p=0.33 to obtain a similar percentage and minimize the percentage of “undesired” one sided and synchrony responses." What do you mean by that? Select for further investigation? Are all simulations going forward using this probability? | Probability p=0.33 was used in all simulations because CNS models with this probability generate swimming in 52% of responses to stimulation and it matches the percentage of swimming responses in experiments (~50%). In addition, for this probability one-sided and synchrony activities are rare across simulation trials (< 5%). We adjusted text to explain these details. |
| Figure 4, legends for c and d, and legends for e and f are swapped | Sorry. We adjusted legends. |
| Page 22, 3rd para: please tone down language like this: “explains how the decision to swim is made”. Your model offers a possible mechanism and should be seen as a prediction. Experimental work is now necessary to test the hypotheses you put forward. Same for this sentence just a few lines down “our modelling reveals the hindbrain neuronal mechanisms of decision making and swimming initiation”. Make sure you edit other sections in the text that overstate your results in this way. | We completely agree with these comments and adjusted the text accordingly. For example: “Our tadpole *CNS model* with realistic connectivity at neuron- and synapse-level resolution offers an explanation about how the decision to swim is made…”. Another example: “Moreover, our modelling allows us to propose hindbrain neuronal mechanisms of decision making and swimming initiation…”. |
| Page 23 2nd para: “measured in microns” your axes in Fig 4 for example are labeled with mm. is it micrometer or mm? | In Fig. 4 we use millimeters (mm) to fit the labels into a small space along the vertical axes. In Results section we use mm but in the Methods (as in our previous publications) cell positions are given in microns. We chose to use microns in the Methods section because we intensively cite the relevant papers where microns have been used. We added a note at the beginning of the Methods section to clarify this matter. |
| Page 25, lower third, there seems to be something missing before “ ” | Thank you for this comment. We have checked Page 25 and adjusted formulas. |
| The Methods section is missing a description of the biomechanical model | We added a new section to METHODS to describe biomechanical model. |
| Code wasn’t available to me, so I can not review its appropriateness. | The code of the CNS model is available from ModelDB repository at <http://modeldb.yale.edu/267146>. For now, it is available privately for the reviewers using the access code **tadpoleplos**. Following Model DB policies, the code will be made available after publication. The code for the VT model developed in Cybernetics is available at [https://github.com/a-palyanov/sibernetic-vt](https://eur03.safelinks.protection.outlook.com/?url=https%3A%2F%2Fgithub.com%2Fa-palyanov%2Fsibernetic-vt&data=04%7C01%7CR.M.Borisyuk%40exeter.ac.uk%7C0ad2563ea8b34860a6ab08d98bdf0224%7C912a5d77fb984eeeaf321334d8f04a53%7C0%7C0%7C637694612924941123%7CUnknown%7CTWFpbGZsb3d8eyJWIjoiMC4wLjAwMDAiLCJQIjoiV2luMzIiLCJBTiI6Ik1haWwiLCJXVCI6Mn0%3D%7C1000&sdata=HCGcsHn7jlOBG%2Bb%2ByE5llpPblEuKnuwNXS%2BRSoPZqKM%3D&reserved=0) |
