# Supplementary material for: From decision to action: Detailed modelling of frog tadpoles reveals neuronal mechanisms of decision-making and reproduces unpredictable swimming movements in response to sensory signals
Source: PLoS Comput Biol. 2021 Dec 13;17(12):e1009654. doi: 10.1371/journal.pcbi.1009654 (PMC8699619; doi:10.1371/journal.pcbi.1009654)
Supplement: Attachment — Submitted filename: second revision reviewers_responses.docx [file pcbi.1009654.s010.docx]

Dear Editors,

Thank you for consideration of our paper for publication in PLOS Computational Biology and to the reviewers for their comments on the manuscript. Please find below our responses to comments of reviewer #3.

| The text still contains numerous grammatical errors. It’s readable, but I wanted to point this out to make you aware in case you have someone else who could look over the text for you again. | Thank you for this note. The grammar has been carefully checked by native speakers. |
| --- | --- |
| My comment #6 (Figure 1): you added hdIN in the figure legend, but there are no hdIN in the figure. If you use two different terms for them in the text, then they should also be represented in the schematic. | Thank you for the comment. For descending interneurons we introduce the abbreviation “dIN”. To simplify explanations we find it useful to introduce another abbreviation ”hdIN” for the descending interneurons in the hind brain. At the first appearance of this abbreviation we explain: **Note**: The population of descending interneurons (*dIN*) is distributed along the body from the hindbrain to tail. We use the abbreviation *hdIN*s to indicate the population of *dIN* located in the hindbrain. (Page 4, par 1). In addition, we deleted this explanation from the figure legend. |
| My comment #9 (Page 8, 3rd para) was addressed in the response but the changes were not made in the text. | The change has been made: The connections between *hexN*s and other populations are specified as follows:…” (Page 8, par 3). |
| My comment #10 (Page 8, 5th para): Your response to me was much clearer than what you added in the method section. I suggest you add a similar explanation to the paper. | We added the following text: “The structure and all parameter values of the physiological model, which generates the functional behaviour, are fixed. To run the physiological model we generate a connectome (adjacency matrix of connections) using the probabilistic algorithms and “project” the connectome to the physiological model. The probabilistic algorithms include random components, therefore, if we repeat the algorithm with unchangeable parameter values, the resulting connectome will differ from all others. We generated many (about 1000) connectomes and, remarkably, all of them produced responses corresponding to the ones found in experiments and described in this paper. “ (Page 28, par 3). In addition we added: **Note**: Using different connectomes, we do not change parameters of the physiological model (see Methods). (Page 8, par 5). |
